# The effectiveness of hysteroscopy for the treatment of cesarean scar pregnancy: a retrospective cohort study

**DOI:** 10.1186/s12884-024-06344-y

**Published:** 2024-02-21

**Authors:** Xinxin You, Yan Ruan, Shouxiang Weng, Chenya Lin, Meifu Gan, Feng Qi

**Affiliations:** Department of Gynecology and Obstetrics, Taizhou Hospital of Zhejiang Province, Zhejiang University, Linhai, Zhejiang Province China

**Keywords:** Cesarean scar pregnancy, Hysteroscopy, Direct gestational tissue removal, Vacuum suction, Serum β-hCG

## Abstract

**Background:**

Cesarean scar pregnancy (CSP) is a long-term complication of cesarean section characterized by the localization of a subsequent gestational sac within the scar area or niche developed as a result of a previous cesarean section. Its incidence has increased substantially because of the high global cesarean section rate in recent decades. Several surgical and drug treatments exist for this condition; however, there is currently no optimal treatment. This study compared the effectiveness of direct hysteroscopic removal of the gestational tissue and hysteroscopy combined with vacuum suction for the treatment of CSP.

**Methods:**

From 2017 to 2023, 521 patients were diagnosed with CSP at our hospital. Of these patients, 45 underwent hysteroscopy. Among them, 28 underwent direct hysteroscopic removal (hysteroscopic removal group) and 17 underwent hysteroscopy combined with vacuum suction (hysteroscopic suction group). The clinical characteristics and outcomes of the hysteroscopic removal group and hysteroscopic suction group were analyzed.

**Results:**

Among the 45 patients, the amount of bleeding and hospitalization cost were significantly higher in the hysteroscopic removal group than in the hysteroscopic suction group (33.8 mL vs. 9.9 mL, *P* < 0.001; and 8744.0 yuan vs. 5473.8 yuan, *P* < 0.001; respectively). The operation time and duration of hospitalization were significantly longer in the hysteroscopic removal group than in the hysteroscopic suction group (61.4 min vs. 28.2 min, *P* < 0.001; and 3.8 days vs. 2.4 days, *P* = 0.026; respectively). Three patients in the hysteroscopic removal group had uterine perforation and received laparoscopic repair during operation. No complications occurred in the hysteroscopic suction group. One patient in the hysteroscopic removal group received ultrasound-guided suction curettage due to postoperative moderate vaginal bleeding, and one patient in the hysteroscopic suction group received ultrasound-guided suction curettage due to postoperative gestational residue and elevated serum beta-human chorionic gonadotropin levels. Reproductive function was preserved in all patients.

**Conclusions:**

Hysteroscopy is an effective method for treating CSP. Compared with direct hysteroscopic removal, hysteroscopy combined with vacuum suction is more suitable for CSP. However, multicenter prospective studies with large sample sizes are required for verification of these findings.

**Supplementary Information:**

The online version contains supplementary material available at 10.1186/s12884-024-06344-y.

## Background

Cesarean scar pregnancy (CSP) is a long-term complication of cesarean section characterized by the localization of a subsequent gestational sac (GS) within the scar area or niche developed as a result of a previous cesarean Sects [1–3]. The true incidence of CSP is unknown; however, the estimated incidence of CSP in a single center has been reported to be between 1:1800 and 1:2656 [4, 5]. The incidence of CSP has increased substantially due to the high global cesarean section rate in recent decades. As a result, this disease has garnered increasing attention from researchers [6, 7]. However, the clinical manifestations of CSP vary and include many asymptomatic cases, making a timely CSP diagnosis difficult [8]. In addition, expectant CSP treatment is prone to severe maternal complications such as massive hemorrhage, placenta accreta spectrum, and uterine rupture. Given these significant risks, pregnancy termination after CSP diagnosis is recommended [8–10]. Several surgical and drug treatments are available for this condition [11–14], but there is no optimal treatment to date.

We hereby summarize our hospital’s experience in treating CSP by hysteroscopy and compare the effectiveness of direct hysteroscopic removal and hysteroscopy combined with vacuum suction for the treatment of CSP.

## Methods

### Patients

**Fig. 1:**
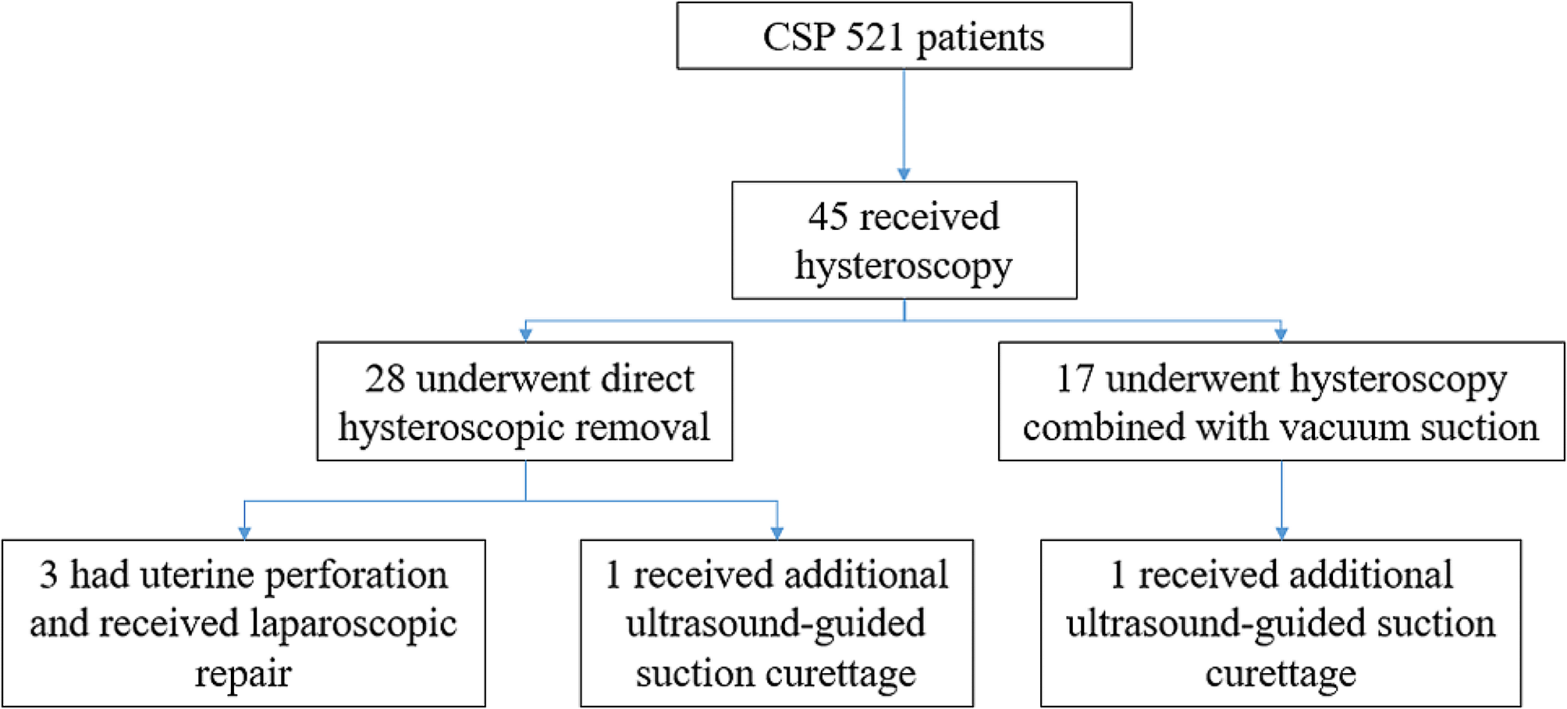
Flow diagram of data collection

We retrospectively analyzed the data of 521 women with CSP admitted to our hospital between 2017 and 2023. After extensive consultation, 45 women underwent hysteroscopy, and written informed consent was obtained before treatment (Fig. 1). The inclusion criteria were as follows: 1) in addition to a history of cesarean delivery and a positive pregnancy test result, CSP diagnosis established using the following transvaginal ultrasound criteria [14–16]: (a) absence of a GS in the cervical canal or uterine cavity; (b) presence of a GS in the original cesarean scar; (c) thinner or even absent myometrium of the anterior wall of the uterus at the attachment site of the GS; (d) low-resistance and high-speed blood flow signals around the GS; and (e) no displacement of the GS when the transvaginal probe was applied to the uterus; 2) stable vital signs; 3) no contraindications for hysteroscopy, such as acute inflammation or severe heart, liver, lung, and kidney disease; and 4) availability of complete medical records and hysteroscopy and ultrasonic data. Patients were followed up through outpatient return visits and telephone consultations. This study was approved by the Ethics Council of Taizhou Hospital (K20190117). The requirement for informed consent was waived because of the retrospective study design.

Among the 45 patients who underwent hysteroscopy, 28 underwent direct hysteroscopic removal (hysteroscopic removal group) and 17 underwent hysteroscopy combined with vacuum suction (hysteroscopic suction group). The effects of the two treatments were compared.

### Procedures

#### Direct hysteroscopic removal

**Fig. 2:**
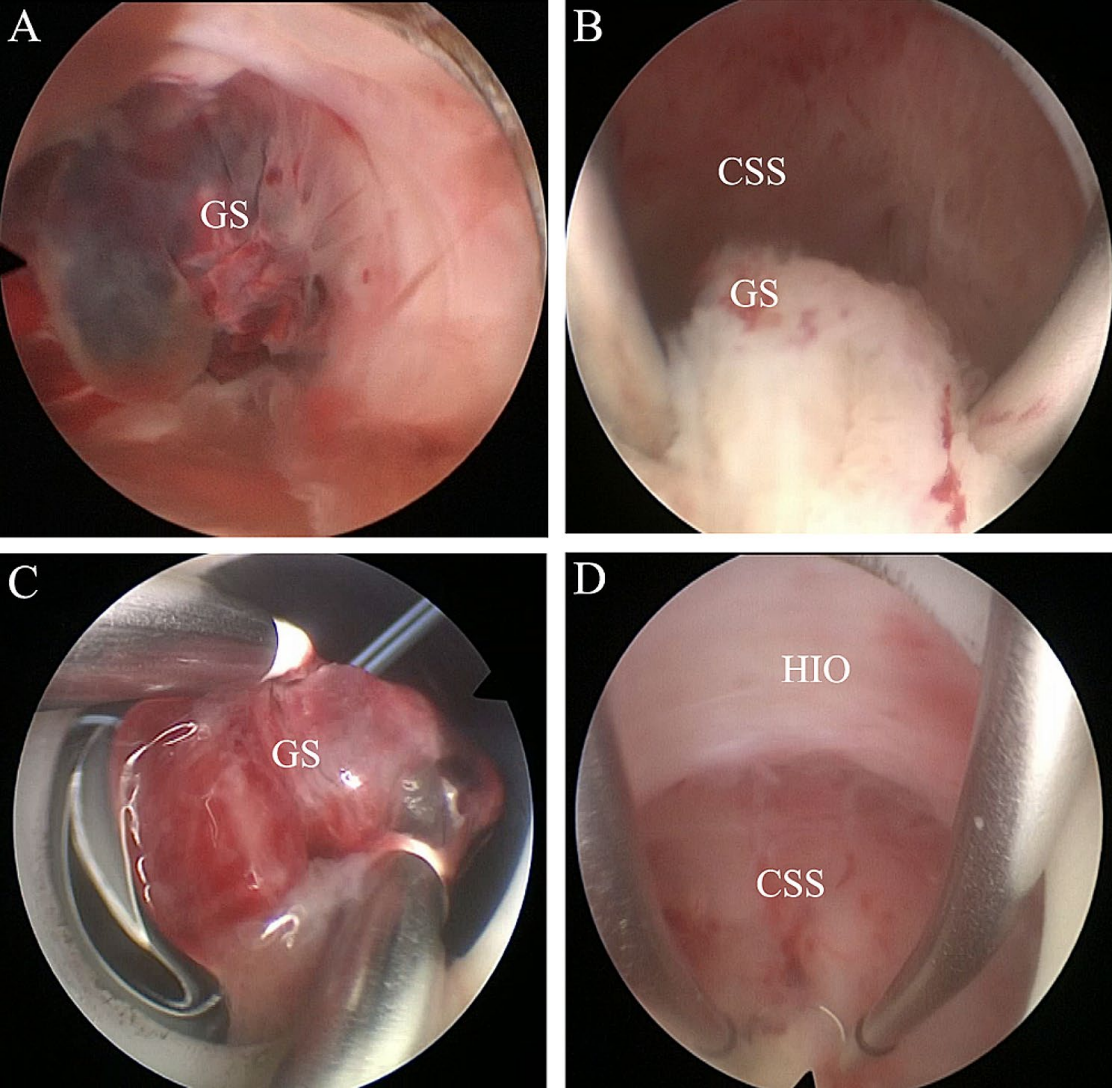
**A**, Hysteroscopic view of cesarean scar pregnancy before hysteroscopic removal. **B** and **C**, An electric loop was used to sweep the gestational tissue bluntly. **D**, Hysteroscopic views of the lower uterine segment confirming no remnants after hysteroscopic resection. CSS, cesarean section scar; GS, gestational sac; HIO, histological internal ostium

Under general anesthesia, patients were placed in the lithotomy position. First, the cervix was dilated to 9.5 cm using a cervical dilator. Next, the uterine cavity was explored with a 26 F hysteroscope with a 30° lens to confirm that the gestational tissue was implanted at the site of the cesarean section scar in the isthmus. Then, after a cervical intramuscular injection of pituitrin 6 U to reduce the risk of bleeding, an electric loop was used to bluntly sweep the gestational tissue. A coagulation current of 70 W was used for hemostasis. Finally, the uterine cavity, scar defect, and cervical canal were thoroughly inspected to ensure they were free of remnants (Fig. 2 and Additional file 1). After the operation, an intravenous drip of oxytocin 10 U was administered to promote uterine contractions, and the samples were sent for pathological examination to confirm the diagnosis.

#### Hysteroscopy combined with vacuum suction

Direct hysteroscopic removal of CSP was performed in our hospital before 2021. However, because the view became easily blurred when gestational tissue was removed directly using an electric loop, which may lead to uterine perforation and a longer operation time, CSP has been treated using hysteroscopy combined with vacuum since 2021. After CSP was confirmed by hysteroscopy (as described above) and a cervical intramuscular injection of pituitrin 6 U was administered, we performed vacuum suction under the guidance of an abdominal ultrasound. Finally, hysteroscopy was performed to confirm the absence of remnants in the uterine cavity and isthmus, followed by electrocoagulation for hemostasis (Additional file 2). After the operation, an intravenous drip of oxytocin 10 U was used to promote uterine contractions, and the samples were sent for pathological examination.

#### Treatment assessment and follow-up

Transvaginal ultrasound and serum beta-human chorionic gonadotropin (β-hCG) monitoring were performed weekly until no residue was found and serum β-hCG levels were normal. Complications were defined as vaginal bleeding of more than 200 mL and uterine perforation during treatment. If the serum β-hCG levels decreased by less than 50% in the first week post-surgery or the volume of vaginal bleeding was more than the usual menstrual volume, additional treatment was administered, including balloon compression, ultrasound-guided vacuum suction, and methotrexate injection. The amount of bleeding, operation time, duration of hospitalization, hospitalization cost, and days to β-hCG resolution were recorded.

### Data analysis

Data are shown as mean ± standard deviation for continuous and ordinal variables and as percentage and absolute counts for categorical variables. Assuming a normal distribution, we used an independent-sample t-test (two-tailed) to compare the values of continuous variables. The χ² or Fisher’s exact test was applied to compare the values of categorical variables. We used a nonparametric test (Mann-Whitney U-test) for non-normally distributed data. SPSS 23.0 (IBM Corp., Armonk, NY, USA) was used for the statistical analysis. *P* < 0.05 was considered statistically significant.

## Results

Among the 45 patients who underwent hysteroscopy, 30 had vaginal bleeding, 3 had hypogastralgia, 1 had vaginal bleeding combined with hypogastralgia, and 11 were asymptomatic. Twenty-five patients were referred to our hospital, as our hospital is a tertiary hospital in this area. Among these 25 patients, eight had been misdiagnosed with an intrauterine pregnancy and had undergone an induced abortion. Three underwent ultrasound-guided suction curettage after diagnosis. These 11 patients were transferred to our hospital due to retained products of conception. The remaining 14 patients were transferred to our hospital after CSP diagnosis.

**Fig. 3:**
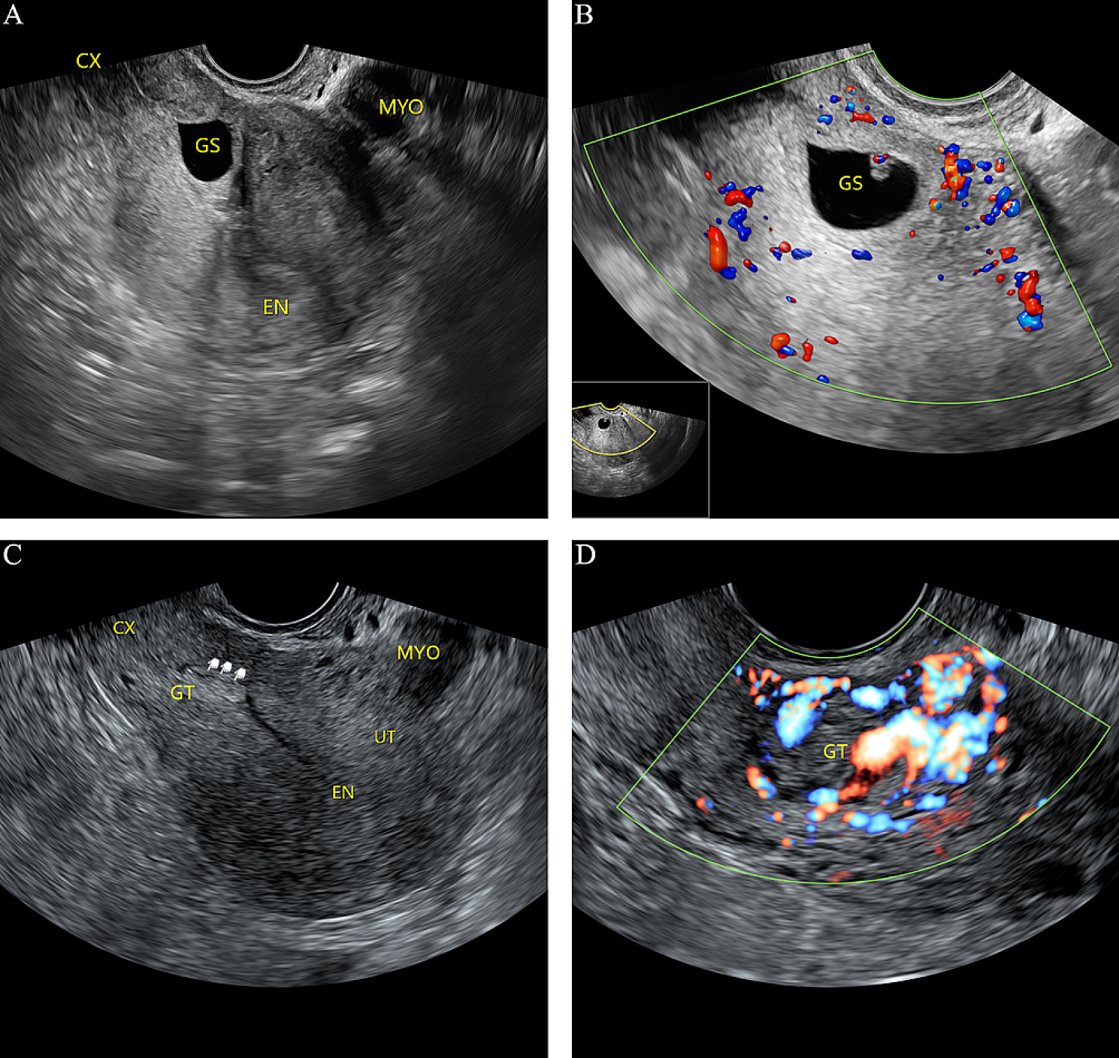
Transvaginal ultrasound image of a cesarean scar pregnancy at 50 postmenstrual days. **A**, Image showing empty uterine cavity and cervical canal, with the gestational sac visible in the lower uterine segment. **B**, Color power Doppler image showing vascularity in the lower uterine segment. **C** and **D**, Image showing gestational residue in the lower uterine segment, with abundant blood flow signal 2 weeks after hysteroscopy. EN, endometrium; MYO, myoma; GS, gestational sac; CX, cervix; GT, gestational residue

A comparison of clinical characteristics and treatment results between the hysteroscopic removal and suction groups is shown in Table 1. The two groups showed no significant differences in age, time from previous cesarean section, percentage of patients transferred from other hospitals, clinical symptoms, trial of labor, number of cesarean sections, serum β-hCG levels, gestational age, gestational sac diameter, fetal cardiac activity, and myometrium thickness. However, the amount of bleeding and hospitalization cost were significantly higher in the hysteroscopic removal group than in the hysteroscopic suction group (33.8 mL vs. 9.9 mL, *P* < 0.001; and 8744.0 yuan vs. 5473.8 yuan, *P* < 0.001; respectively). The operation time and duration of hospitalization were significantly longer in the hysteroscopic removal group than in the hysteroscopic suction group (61.4 min vs. 28.2 min, *P* < 0.001; and 3.8 days vs. 2.4 days, *P* = 0.026; respectively). Three patients (11%, 3/28) from the hysteroscopic removal group had uterine perforation and received laparoscopic repair during operation; no complications occurred in the hysteroscopic suction group. One patient from the hysteroscopic removal group received ultrasound-guided suction curettage due to postoperative moderate vaginal bleeding, and one patient who underwent hysteroscopy combined with vacuum suction received ultrasound-guided suction curettage due to postoperative gestational residue and elevated serum β-hCG levels (Fig. 3); however, these differences were not statistically significant. Pathological examination showed villous tissue in all patients. Reproductive function was preserved for all patients. In addition, there was no significant difference between the two groups concerning the duration of hospitalization and days to β-hCG resolution.

**Table 1:** Characteristics and results of women treated with direct hysteroscopic removal or hysteroscopy and vacuum suction

Characteristic		Hysteroscopic removal group	Hysteroscopic suction group	*P*-value
No. of patients		28	17	N/A
Age (years)		34.5 ± 5.1	33.3 ± 5.7	0.453
Time from previous CS (years)		6.0 ± 4.7	6.4 ± 4.5	0.825
Trial of labor (%)		0.0 (0)	11.8 (2)	0.137
Transferred from other hospitals (%)		60.7 (17)	47.1 (8)	0.537
Clinical symptoms	Vaginal bleeding (%)	64.3 (18)	76.5 (13)	0.513
	hypogastralgia (%)	10.7 (3)	5.9 (1)	1.000
	asymptomatic (%)	32.1 (9)	11.8 (2)	0.165
No. of cesarean		1.8 ± 0.5	1.6 ± 0.6	0.351
Serum β-hCG levels (IU/L)		34347.3 ± 43597.2	30412.4 ± 45249.4	0.774
Gestational age (days)		55.1 ± 16.3	50.5 ± 20.6	0.410
Gestational sac diameter (mm)		29.1 ± 14.6	23.1 ± 11.8	0.161
Fetal cardiac activity (%)		35.7 (10)	29.4 (5)	1.000
Thickness of myometrium (mm)		2.7 ± 1.5	2.3 ± 1.4	0.752
Complication rate (%)		10.7 (3/28)	0.0 (0/10)	0.552
Additional treatment rate (%)		3.6 (1/28)	10.0 (1/10)	0.462
Amount of bleeding (ml)		33.8 ± 29.0	9.9 ± 11.6	**< 0.001**
Operation time (minutes)		61.4 ± 30.5	28.2 ± 8.5	**< 0.001**
Hospitalization cost (yuan)		8744.0 ± 3148.7	5473.8 ± 1312.8	**< 0.001**
Duration of hospitalization (days)		3.8 ± 2.2	2.4 ± 1.6	**0.026**
Days to β-hCG resolution (days)		26.6 ± 14.1	21.0 ± 8.0	0.098

CS, cesarean section; β-hCG, β-human chorionic gonadotropin

## Discussion

Currently, the main treatment methods of CSP include drug therapy and surgical treatment. Drug therapies include intramuscular injection of methotrexate (MTX) and ultrasound-guided local injection of MTX, absolute alcohol, or potassium chloride. Surgical treatments include ultrasound-guided uterine aspiration, transabdominal, laparoscopic, hysteroscopic, and transvaginal resection of gestational tissue or hysterectomy. In addition, uterine artery embolization, lauromacrogol local injection, balloon compression, and high-intensity focused ultrasound can be used in combination with drugs or surgery to treat CSP [11, 12, 17–19]. Hysteroscopy is one of the recommended treatments for CSP [14, 20]. Under direct visualization, CSP can be diagnosed accurately, and the possibility of gestational trophoblastic neoplasia and inevitable abortion can be excluded, especially when the gestational residue of CSP continues to grow in the lower segment of the anterior uterine wall and forms a mass after incomplete abortion or suction curettage. Ultrasonography images are similar to those of gestational trophoblastic neoplasia [16, 21]. For instance, there is no obvious boundary with the muscular layer, the local muscular layer is absent or thinning, the local blood flow signal is extremely rich, and high-speed and low-resistance blood flow can be detected. Hysteroscopy can help in making a definitive diagnosis through direct visualization and pathological sampling. Hysteroscopy for the treatment of CSP was gradually performed in our hospital after we learned hysteroscopic techniques at the Beijing International Hysteroscopy Center and studied the literature on successful hysteroscopic treatment of CSP [11, 22, 23]; thus, only 45 patients with CSP were treated with hysteroscopy in our study. Among them, eight patients were misdiagnosed with intrauterine early pregnancy and received early induced abortion; the misdiagnosis rate was 17.8%, similar to that in previous studies [8, 12]. Eleven patients were transferred to our hospital because of gestational residue, and all were successfully diagnosed and treated using hysteroscopy. In addition, hysteroscopy can confirm the absence of remnants in the GS and can be used to perform electrocoagulation hemostasis after treatment.

Hysteroscopy is typically used to treat CSP via direct resection [23–25]. However, in treating CSP using hysteroscopy, we found that the view is easily blurred when the gestational tissue is removed directly using an electric loop, which may lead to uterine perforation. Moreover, a longer operation time increases irrigation with saline, which is used for uterine distension, leading to an increased risk of water intoxication and heart failure. Therefore, we attempted to treat CSP using hysteroscopy combined with vacuum suction. The results showed no complications such as uterine perforation or massive vaginal bleeding in women treated with hysteroscopy combined with vacuum suction. In addition, compared with direct hysteroscopic removal, hysteroscopy combined with vacuum suction had a significantly shorter operative time and lower intraoperative blood loss, and hospitalization costs were significantly reduced. Therefore, hysteroscopy combined with vacuum suction seems to be more effective than hysteroscopic removal for treating CSP.

Our study has some limitations. First, the cesarean section diverticulum after hysteroscopy is not repaired or is even larger than before due to the implantation and growth of pregnancy tissue, which may lead to subsequent menostaxis, infertility, recurrent CSP, and uterine rupture after a second pregnancy. Therefore, further studies on fertility after CSP treated with hysteroscopy are needed. Second, this is a retrospective study with a small sample from a single center, and there was the possibility of bias in the division between hysteroscopic removal or hysteroscopy combined with vacuum suction, which reduces the overall robustness of the analysis; therefore, a multicenter prospective study enrolling a larger sample of patients with CSP is needed.

## Conclusions

Hysteroscopy is an effective method for treating CSP. Compared with direct hysteroscopic removal, hysteroscopy combined with vacuum suction is more suitable for CSP. However, future prospective studies with multiple centers and large sample sizes are required for verification.

### Electronic supplementary material

Below is the link to the electronic supplementary material.

Additional file 1: Direct hysteroscopic removal for the treatment of CSP

Additional file 2: Hysteroscopy combined with vacuum suction for the treatment of CSP

## Data Availability

All data generated or analyzed during this study are included in this published article. Further inquiries can be directed to the corresponding author.

## Abbreviations

CSP: Cesarean scar pregnancy
β-hCG: beta human chorionic gonadotropin
GS: Gestational sac
MTX: Methotrexate

## References

[1] Vial Y, Petignat P, Hohlfeld P (2000). Pregnancy in a cesarean scar. Ultrasound Obstet Gynecol.

[2] Godin PA, Bassil S, Donnez J (1997). An ectopic pregnancy developing in a previous caesarian section scar. Fertil Steril.

[3] Ash A, Smith A, Maxwell D (2007). Caesarean scar pregnancy. BJOG.

[4] Jurkovic D, Hillaby K, Woelfer B, Lawrence A, Salim R, Elson C (2003). First-trimester diagnosis and management of pregnancies implanted into the lower uterine segment cesarean section scar. Ultrasound Obstet Gynecol.

[5] Seow KM, Huang LW, Lin YH, Lin MY, Tsai YL, Hwang JL (2004). Cesarean scar pregnancy: issues in management. Ultrasound Obstet Gynecol.

[6] Riaz RM, Williams TR, Craig BM, Myers DT (2015). Cesarean scar ectopic pregnancy: imaging features, current treatment options, and clinical outcomes. Abdom Imaging.

[7] Gibbons L, Belizan JM, Lauer JA, Betran AP, Merialdi M, Althabe F (2012). Inequities in the use of cesarean section deliveries in the world. Am J Obstet Gynecol.

[8] Rotas MA, Haberman S, Levgur M (2006). Cesarean scar ectopic pregnancies: etiology, diagnosis, and management. Obstet Gynecol.

[9] Maymon R, Halperin R, Mendlovic S, Schneider D, Vaknin Z, Herman A (2004). Ectopic pregnancies in caesarean section scars: the 8 year experience of one medical centre. Hum Reprod.

[10] Osborn DA, Williams TR, Craig BM (2012). Cesarean scar pregnancy: Sonographic and magnetic resonance imaging findings, complications, and treatment. J Ultrasound Med.

[11] Birch Petersen K, Hoffmann E, Rifbjerg Larsen C, Nielsen HS (2016). Cesarean scar pregnancy: a systematic review of treatment studies. Fertil Steril.

[12] Timor-Tritsch IE, Monteagudo A (2012). Unforeseen consequences of the increasing rate of cesarean deliveries: early placenta accreta and cesarean scar pregnancy. A review. Am J Obstet Gynecol.

[13] Zhou W, Feng X, Yu J, Chai Z, Zheng L, Qi F (2022). The efficacy of different treatments for type 2 cesarean scar pregnancy. Fertil Steril.

[14] Miller R, Gyamfi-Bannerman C (2022). Society for maternal-fetal medicine consult series #63: cesarean scar ectopic pregnancy. Am J Obstet Gynecol.

[15] Timor-Tritsch IE, Monteagudo A, Santos R, Tsymbal T, Pineda G, Arslan AA (2012). The diagnosis, treatment, and follow-up of cesarean scar pregnancy. Am J Obstet Gynecol.

[16] Family planning group of obstetrics and gynecology branch of the chinese medical association (2016). Expert consensus on the diagnosis and management of cesarean scar pregnancy. Chin J Obstet Gynecol.

[17] Xiao J, Zhang S, Wang F, Wang Y, Shi Z, Zhou X (2014). Cesarean scar pregnancy: noninvasive and effective treatment with high-intensity focused ultrasound. Am J Obstet Gynecol.

[18] Chai ZY, Yu L, Liu MM, Zhu TW, Qi F (2018). Evaluation of the efficacy of ultrasound-guided local lauromacrogol injection combined with aspiration for cesarean scar pregnancy: a novel treatment. Gynecol Obstet Invest.

[19] Wu Q, Liu X, Zhu L, Zhu Y, Mei T, Cao S (2020). Clinical assessment of ultrasound-guided local lauromacrogol injection combined with curettage and hysteroscopy for cesarean scar pregnancy. Front Pharmacol.

[20] Tang Q, Qin Y, Zhou Q, Tang J, Zhou Q, Qiao J (2021). Hysteroscopic treatment and reproductive outcomes in cesarean scar pregnancy: experience at a single institution. Fertil Steril.

[21] Yu XL, Zhang N, Zuo WL (2011). Cesarean scar pregnancy: an analysis of 100 cases. Chin J Obstet Gynecol.

[22] Pan Y, Liu MB (2017). The value of hysteroscopic management of cesarean scar pregnancy: a report of 44 cases. Taiwan J Obstet Gynecol.

[23] Mollo A, Conforti A, Alviggi C, De Placido G (2014). Successful direct bipolar resection of 6th week cesarean scar pregnancy: case report and literature review. Eur J Obstet Gynecol Reprod Biol.

[24] Chueh HY, Pai AH, Su YY, Hsu CC, Chang FY, Yen CF (2022). Hysteroscopic removal, with or without laparoscopic assistance, of first-trimester cesarean scar pregnancy. Fertil Steril.

[25] Wang DB, Chen YH, Zhang ZF, Chen P, Liu KR, Li Y (2014). Evaluation of the transvaginal resection of low-segment cesarean scar ectopic pregnancies. Fertil Steril.

